# Dual Immunoglobulin Domain-Containing Cell Adhesion Molecule Increases Early in Renal Tubular Cell Injury and Plays Anti-Inflammatory Role

**DOI:** 10.3390/cimb46030115

**Published:** 2024-02-26

**Authors:** Jin Han, Ju-Min Yook, Se-Hyun Oh, Yu Kyung Chung, Hee-Yeon Jung, Ji-Young Choi, Jang-Hee Cho, Sun-Hee Park, Chan-Duck Kim, Yong-Lim Kim, Seungwoo Han, Jeong-Hoon Lim

**Affiliations:** 1 Laboratory for Arthritis and Cartilage Biology, Research Institute of Aging and Metabolism, Kyungpook National University, Daegu 41404, Republic of Korea; jinhan6628@gmail.com (J.H.); kiefe73@gmail.com (S.H.); 2 Cell & Matrix Research Institute, School of Medicine, Kyungpook National University, Daegu 41944, Republic of Korea; ttily@nate.com (S.-H.O.); jh-cho@knu.ac.kr (J.-H.C.); ylkim@knu.ac.kr (Y.-L.K.); 3 Division of Nephrology, Department of Internal Medicine, School of Medicine, Kyungpook National University, Kyungpook National University Hospital, Daegu 41944, Republic of Korea; jumin18@naver.com (J.-M.Y.); ykyk8904@naver.com (Y.K.C.); jyss1002@hanmail.net (J.-Y.C.); sh-park@knu.ac.kr (S.-H.P.); drcdkim@knu.ac.kr (C.-D.K.); 4 Division of Rheumatology, Department of Internal Medicine, School of Medicine, Kyungpook National University, Kyungpook National University Hospital, Daegu 41944, Republic of Korea

**Keywords:** DICAM, acute kidney injury, diagnostic marker, anti-inflammation, renal tubular injury

## Abstract

Dual immunoglobulin domain-containing cell adhesion molecule (DICAM) is a type I transmembrane protein that presents in various cells including renal tubular cells. This study evaluated the expression and protective role of DICAM in renal tubular cell injury. HK-2 cells were incubated and treated with lipopolysaccharide (LPS, 30 μg/mL) or hydrogen peroxide (H_2_O_2_, 100 μM) for 24 h. To investigate the effect of the gene silencing of DICAM, small interfering RNA of DICAM was used. Additionally, to explain its role in cellular response to injury, DICAM was overexpressed using an adenoviral vector. DICAM protein expression levels significantly increased following treatment with LPS or H_2_O_2_ in HK-2 cells. In response to oxidative stress, DICAM showed an earlier increase (2–4 h following treatment) than neutrophil gelatinase-associated lipocalin (NGAL) (24 h following treatment). DICAM gene silencing increased the protein expression of inflammation-related markers, including IL-1β, TNF-α, NOX4, integrin β1, and integrin β3, in H_2_O_2_-induced HK-2 cell injury. Likewise, in the LPS-induced HK-2 cell injury, DICAM knockdown led to a decrease in occludin levels and an increase in integrin β3, IL-1β, and IL-6 levels. Furthermore, DICAM overexpression followed by LPS-induced HK-2 cell injury resulted in an increase in occludin levels and a decrease in integrin β1, integrin β3, TNF-α, IL-1β, and IL-6 levels, suggesting an alleviating effect on inflammatory responses. DICAM was elevated in the early stage of regular tubular cell injury and may protect against renal tubular injury through its anti-inflammatory properties. DICAM has a potential as an early diagnostic marker and therapeutic target for renal cell injury.

## 1. Introduction

Acute kidney injury (AKI) indicates a clinical disease entity characterized by an abrupt decline in renal function that causes impairments in waste product excretion, fluid balance regulation, and electrolyte homeostasis maintenance [1,2,3]. AKI is common in 1 in 10 hospitalized patients and 3 in 10 critically ill patients [4,5]. Moreover, AKI incidence is increasing annually [6]. AKI is a critical medical condition that significantly raises patient mortality rates [1].

AKI pathogenesis is not restricted to a single triggering event. Instead, it occurs from multiple potential causes. For example, reduced renal blood flow, as observed in ischemic injuries, to direct tissue damage is instigated by inflammatory processes. Furthermore, AKI occurrence is affected by further downstream, toxin exposure; systemic infections; and certain pharmacological agents [7]. Considering the broad spectrum of triggering factors and the potential for rapid progression, the early detection and identification of the underlying etiology are of utmost significance in AKI management. Various predictive markers of AKI occurrence, such as neutrophil gelatinase-associated lipocalin (NGAL) and Kidney Injury Molecule-1, have been developed to date. However, these markers have limitations and remain of limited use. Therefore, there is an unmet need to develop markers that can effectively predict AKI at an early stage.

Dual immunoglobulin domain-containing cell adhesion molecule (DICAM), also known as limitrin and matrix remodeling-associated 8, is a type I transmembrane protein with two V-type immunoglobulin domains. Originally identified in the end-feet of astrocytes that form the blood–brain barrier, DICAM has phylogenetic homology with the junctional adhesion molecule family. It is ubiquitously expressed in a variety of cell types, including macrophages, Th17 cells, endothelial cells, epithelial cells, and chondrocytes, and is known to play a role in regulating inflammatory responses, particularly by increasing under stress conditions, but its role in renal cell injury, such as acute kidney injury, remains unclear [8,9,10,11,12,13].

Our previous study showed the DICAM expression in renal proximal tubular cells [9]. In addition, the expression of DICAM was increased in the cisplatin-induced AKI mouse model [14]. The inhibition of DICAM was found to further promote apoptosis and inflammation in the injured kidney in animal experiments, but this has not yet been clearly confirmed in cellular experiments. Furthermore, because the causes of AKI are diverse, the implications of its expression and role, particularly in the context of AKI due to various causes, remain a topic of exploration. This study evaluated the expression dynamics of DICAM in renal proximal tubular cells when subjected to oxidative stress via hydrogen peroxide (H_2_O_2_) and inflammatory stress simulated through lipopolysaccharide (LPS) exposure. This study aimed to provide a foundational understanding of DICAM’s significance in renal cellular responses, thereby offering insights for potential prediction and therapeutic strategies in AKI.

## 2. Materials and Methods

### 2.1. Cell Culture

HK-2 (CRL-2190™) were purchased from the American Type Culture Collection (ATCC; Manassas, VA, USA). HK-2 cells were cultured in RPMI-1640 supplemented with 10% fetal bovine serum, 100 U/mL penicillin, and 100 µg/mL streptomycin. Cells were maintained at 37 °C in a humidified atmosphere containing 5% CO_2_.

### 2.2. Treatment Protocols

For oxidative stress induction, cells were treated with H_2_O_2_ at a 100 μM concentration. For inflammatory stimulation, cells were treated with LPS at a 30 μg/mL concentration. Cell viability was confirmed to determine the treatment concentration (Figure S1). All treatments were performed in serum-free media.

### 2.3. Small Interfering RNA (siRNA) Transfection

DICAM-specific siRNA and non-targeting control siRNA were obtained from Cell Signaling Technology (Danvers, MA, USA). For siRNA transfection, HK-2 cells were seeded in six-well plates and transfected using Lipofectamine RNAiMax (Invitrogen, Paisley, UK) according to the manufacturer’s instructions. The silencing efficacy was verified 6 h post-transfection using Western blotting.

### 2.4. Adenoviral Overexpression

To overexpress DICAM in HK-2 cells, an adenoviral vector expressing DICAM was used. Cells were transduced with the adenoviral vector (100 MOI) 4 h before LPS treatment, thereby allowing for the efficient overexpression of the protein. Recombinant adenovirus (Ad) expressing full-length DICAM (Ad-DICAM) or β-galactosidase (Ad-LacZ) was prepared as previously described [15].

### 2.5. Western Blot Analysis

After treatment, cells were lysed in RIPA buffer containing protease and phosphatase inhibitors. Equal amounts of protein determined using Bradford’s method were separated using SDS-PAGE and transferred onto nitrocellulose membranes. Membranes were blocked in 5% non-fat milk for 1 h at room temperature and incubated with primary antibodies against DICAM (1:1000; Novus Biologicals, Toronto, ON, Canada), occludin (1:1000; Abcam, Cambridge, MA, USA), NGAL (1:1000; Invitrogen, Carlsbad, CA, USA), NADPH Oxidase 4 (NOX4; 1:1000; Abcam), IL-1β (1:1000; Cell Signaling Technology), IL-6 (1:1000; Abcam), TNF-α (1:1000; Abcam), integrin β1 (1:1000; Cell Signaling Technology), integrin β3 (1:1000; Cell Signaling Technology), IL-8 (1:1000; Abcam, Cambridge, MA, USA), and anti-GAPDH (1:2000; Cell Signaling Technology) overnight at 4 °C. Membranes were incubated with horseradish peroxidase-conjugated secondary antibodies after washing. Bands were visualized using an enhanced chemiluminescence system and visualized on an ImageQuant™ LAS 4000 system (GE HealthcareLife Sciences, Tokyo, Japan). The protein band densities were quantified using the Scion Image software (version 4.0, Scion, Frederick, MD, USA).

### 2.6. Quantitative Real-Time RT-PCR

The total RNA was isolated from HK-2 cells using TRIzol (Invitrogen, Carlsbad, CA, USA), and the first-strand cDNA was synthesized using Superscript III reverse transcriptase (Invitrogen). Real-time qPCR was performed using a ViiA™ 7 Real-Time PCR System (Applied Biosystems, Foster City, CA, USA) and SYBR^®^ Green Master Mix (Applied Biosystems). The primers used were specific primer sets [catalase: (forward 5′-CCAAATACTCCAAGGCAAAGGT-3′), (reverse 5′-AACCCGATTCTCCAGCAACA-3′), SOD1: (forward 5′-GCGGAGGTCTGGCCTATAAAG-3′), (reverse 5′-CTGGTTCCGAGGACTGCAA-3′), SOD2: (forward 5′-GCTTGCAAAAAGTAAACCACGAT-3′), (reverse 5′-CCAGGCTTGATGCACATCTTAG-3′), NOX4: (forward 5′-AAGCCAGTCACCATCATTTCG-3′), (reverse 5′-CTTTGACCATTCGGATTTCCA-3′), Bcl2: (forward: 5′-GGGGACGAACTGGACAGTAA-3′), (reverse: 5′-CAGTTGAAGTTGCCGTCAGA-3′), Bax: (forward: 5′-GGGGACGAACTGGACAGTAA-3′), (reverse: 5′-CAGTTGAAGTTGCCGTCAGA-3′), GAPDH: (forward 5′-TTCACCACCATGGAGAAGGCT-3′), (reverse 5′-TGGTTCACACCCATGACGAAC-3′)]. GAPDH was used as a reference control.

### 2.7. Caspase-3 Assay

HK-2 cells were incubated in media containing PBS (control) or H_2_O_2_ or LPS for 2, 4, and 24 hr. At the end of the incubation period, the cells were harvested and prepared for measurements of caspase-3 activity with the use of a Caspase-3 assay kit (Casp-3-C, Sigma, St. Louis, MO, USA).

### 2.8. Statistical Analysis

All data were subjected to statistical evaluation using a Kruskal–Wallis test. To determine the significance of differences between individual groups, post hoc analyses were performed employing Tukey’s multiple comparison tests. A *p*-value of <0.05 was considered statistically significant. All results were presented as means ± standard deviations.

## 3. Results

### 3.1. DICAM Expression in H_2_O_2_ and LPS-Induced Injury of HK-2 Cells

**Figure 1:**
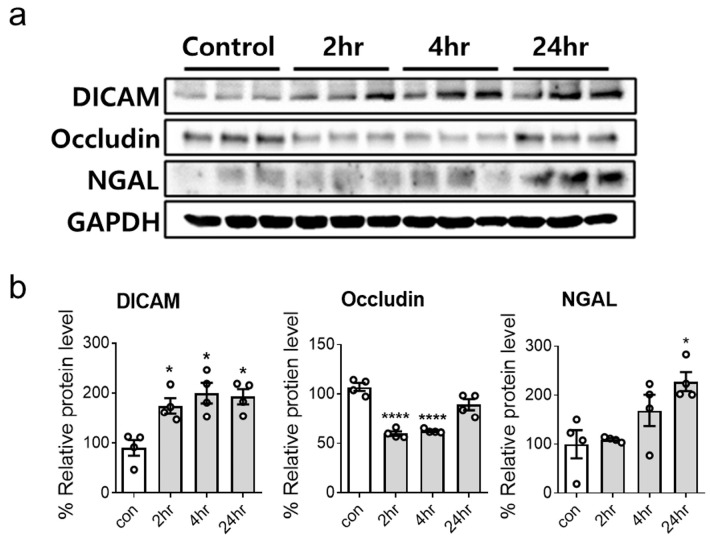
Hydrogen peroxide (H_2_O_2_) treatment increases DICAM protein expression in HK-2 cells. (**a**) Representative Western blot images depict the protein levels of DICAM, occludin, neutrophil gelatinase-associated lipocalin (NGAL), and GAPDH in HK-2 cells following H_2_O_2_ exposure. (**b**) Quantification of the Western blot from (**a**) reveals significant variations in protein expression. * *p* < 0.05 and **** *p* < 0.0001 indicate statistical significance. Abbreviations: DICAM, dual immunoglobulin domain-containing cell adhesion molecule; NGAL, neutrophil gelatinase-associated lipocalin; GAPDH, glyceraldehyde-3-phosphate dehydrogenase.

The temporal expression pattern of DICAM in HK-2 cells under AKI conditions induced by H_2_O_2_ and LPS was analyzed. HK-2 cells exposed to 100 uM H_2_O_2_ showed a significant increase in DICAM expression at 2, 4, and 24 h post-treatment. Concurrently, a decline in occludin levels was observed at 2 and 4 h, with an increase in NGAL expression at the 24 h time point (Figure 1). The results of the H_2_O_2_-induced AKI model suggested that DICAM plays a role during tubular cell injury progression, potentially influencing cellular junction integrity (as indicated by the occludin levels) and kidney injury (indicated by the NGAL levels).

**Figure 2:**
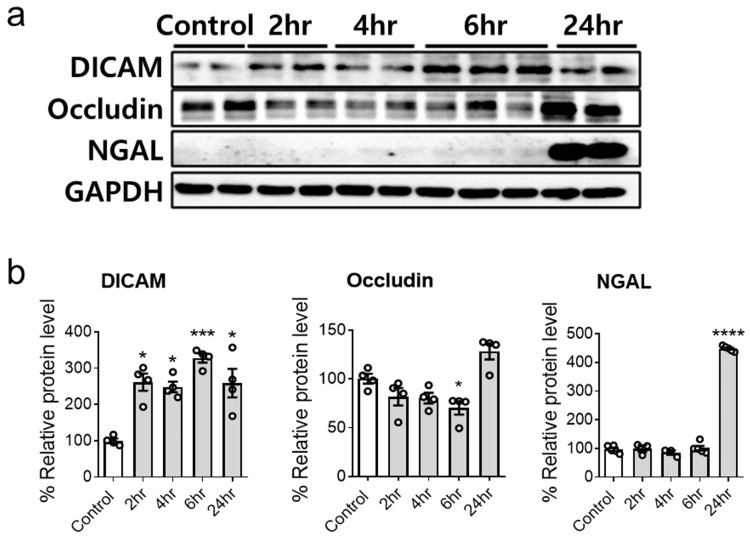
Lipopolysaccharide (LPS) treatment increases DICAM protein expression in HK-2 cells. (**a**) Representative Western blot images depict the protein levels of DICAM, occludin, NGAL, and GAPDH in HK-2 cells following H_2_O_2_ exposure. (**b**) Quantification of the Western blot from (**a**) reveals significant variations in protein expression. * *p* < 0.05, *** *p* < 0.001, and **** *p* < 0.0001 indicate statistical significance. Abbreviations: DICAM, dual immunoglobulin domain-containing cell adhesion molecule; NGAL, neutrophil gelatinase-associated lipocalin; GAPDH, glyceraldehyde-3-phosphate dehydrogenase.

When treated with 30 μg/mL LPS, the HK-2 cells exhibited heightened DICAM expression at 2, 4, 6, and 24 h. Similar to the H_2_O_2_ model, occludin levels decreased at 6 h, whereas NGAL levels increased at 24 h (Figure 2). The response in the LPS model further corroborates the potential role of DICAM in tubular cell injury, indicating its potential consistency across different AKI causes.

### 3.2. Influence of DICAM Deficiency on H_2_O_2_ and LPS-Induced HK-2 Cell Injury

**Figure 3:**
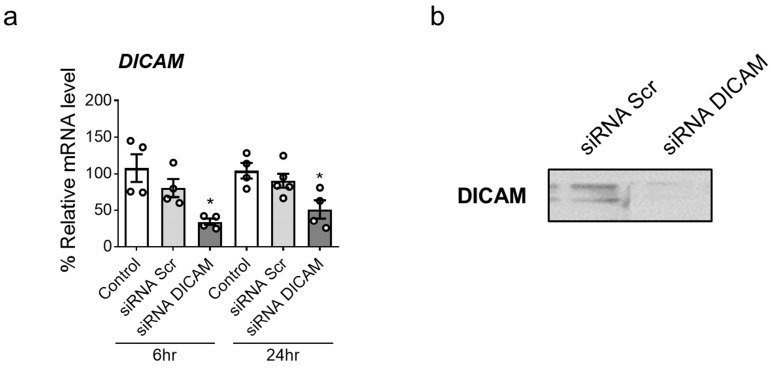
Effective DICAM expression suppression in HK-2 cells using siRNA-targeting DICAM. (**a**) Transfection conditions are established to inhibit DICAM expression. HK-2 cells are transfected with siRNA-targeting DICAM for 6 and 24 h time points. A statistically significant decrease in DICAM expression is observed at 6 and 24 h. Results demonstrate that a 6 h transfection duration is adequate for substantial DICAM suppression. (**b**) Confirmation of DICAM knockdown following 6 h siRNA–DICAM treatment using DICAM antibody and Western blot analysis. * *p* < 0.05 indicates statistical significance. Abbreviations: siRNA, small interfering RNA; Scr, scramble (negative control); DICAM, dual immunoglobulin domain-containing cell adhesion molecule.

Subsequently, we investigated how DICAM expression suppression affected the cellular response to HK-2 cell injury induced by H_2_O_2_ and LPS. Following transfection with siRNA-targeting DICAM, DICAM expression suppression was observed up to 6 h post-transfection (Figure 3). Efficient DICAM knockdown offers the ability to explore its direct implications on AKI responses in subsequent experiments.

**Figure 4:**
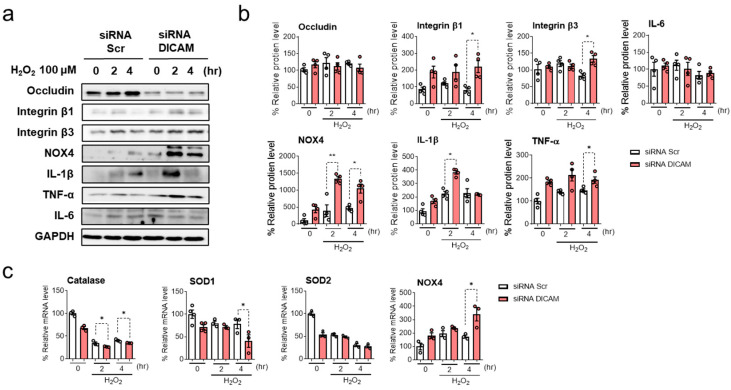
Impact of DICAM gene knockdown on the protein expression of associated markers in H_2_O_2_-induced HK-2 cell injury. (**a**) Following DICAM knockdown using siRNA control and siRNA DICAM, occludin, integrin β1, integrin β3, NOX4, IL-1β, and GAPDH protein levels were assessed at 2 and 4 h following H_2_O_2_ treatment. (**b**) Quantitative analysis of the protein expression levels. (**c**) Antioxidant markers including catalase, SOD1, SOD2, and oxidative stress marker NOX4 were analyzed using real-time RT-qPCR. * *p* < 0.05 and ** *p* < 0.01 indicate statistical significance. Abbreviations: siRNA, small interfering RNA; Scr, scramble (negative control); DICAM, dual immunoglobulin domain-containing cell adhesion molecule; NOX4, NADPH oxidase 4; IL-1β, interleukin-1 beta; TNF-α, tumor necrosis factor-α; IL-6, interleukin 6; GAPDH, glyceraldehyde-3-phosphate dehydrogenase.

In the presence of DICAM knockdown, HK-2 cells exposed to 100 uM H_2_O_2_ showed increases in integrin β1 and integrin β3 levels at 4 h post-treatment (Figure 4). Furthermore, elevated levels of inflammatory cytokines IL-1β at 2 h and TNF-α at 4 h were noted, indicating an accelerated inflammatory response (Figure 4b). Additionally, antioxidant markers including catalase and SOD1 decreased and oxidative stress marker such as NOX4 levels increased following LPS treatment after DICAM knockdown (Figure 4c). The change in these markers suggests that DICAM possesses protective qualities against oxidative stress and inflammation during AKI.

**Figure 5:**
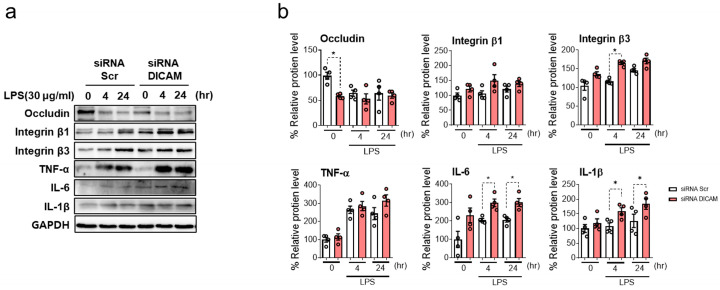
Impact of DICAM gene knockdown on the protein expression of associated markers in LPS-induced HK-2 cell injury. (**a**) Following DICAM knockdown using siRNA control and siRNA DICAM, occludin, integrin β1, integrin β3, TNF-α, IL-1β, and GAPDH protein levels were assessed at 2 and 4 h following LPS treatment. (**b**) Quantitative analysis of the protein expression levels. * *p* < 0.05 indicates statistical significance. Abbreviations: siRNA, small interfering RNA; Scr, scramble (negative control); DICAM, dual immunoglobulin domain-containing cell adhesion molecule; IL-6, interleukin 6; TNF-α, tumor necrosis factor-α; IL-1β, interleukin-1 beta; GAPDH, glyceraldehyde-3-phosphate dehydrogenase.

Following DICAM knockdown, the HK-2 cells treated with 30 μg/mL LPS showed increased integrin β3 levels, particularly at the 4 h time point. Moreover, the IL-6 and IL-1β levels increased at 4 and 24 h following treatment, whereas the occludin levels increased at the baseline (0 h) (Figure 5). These alterations in protein expression on the role of DICAM suggest not only a protective anti-inflammatory effect but also a potential regulatory impact on cell adhesion and integrity during tubular cell injury.

### 3.3. Impact of DICAM Overexpression on LPS-Induced HK-2 Cell Injury

**Figure 6:**
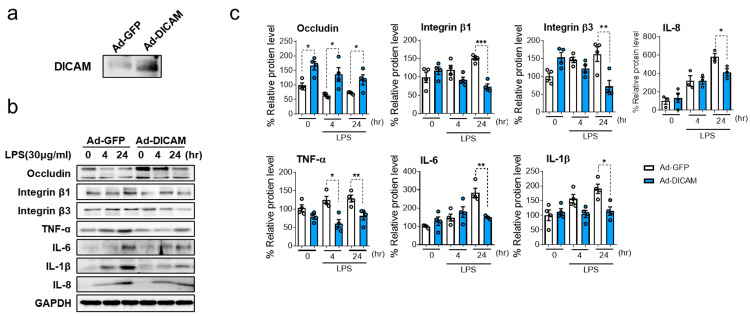
Altered protein expression of associated markers in LPS-induced HK-2 cell injury following DICAM overexpression. (**a**) DICAM overexpression verification following treatment with adenovirus DICAM, as depicted by Western blot analysis. (**b**) Following DICAM overexpression with adenovirus DICAM, cells were exposed to LPS for 4 and 24 h. Subsequent Western blotting was performed, employing antibodies targeting occludin, integrin β1, integrin β3, TNF-α, IL-6, IL-8, and GAPDH. (**c**) Quantitative evaluation of the protein expression patterns presented in panel (**b**). * *p* < 0.05, ** *p* < 0.01, and *** *p* < 0.001 indicate statistical significance. Abbreviations: Ad-GFP, adenovirus expressing green fluorescent protein; Ad-DICAM, adenovirus expressing dual immunoglobulin domain-containing cell adhesion molecule; TNF-α, tumor necrosis factor-α; IL-6, interleukin 6; IL-1β, interleukin-1 beta; GAPDH, glyceraldehyde-3-phosphate dehydrogenase.

Changes in cellular responses were observed when DICAM was overexpressed during LPS-induced HK-2 cell injury. HK-2 cells were transduced with an adenoviral vector bearing the DICAM gene, thereby facilitating its overexpression. Subsequent to ensuring successful overexpression (Figure 6a), the transduced cells were subjected to LPS stress using a 30 μg/mL concentration. When the DICAM was overexpressed, upon exposure to LPS, a marked upregulation in occludin expression was observed at 4 and 24 h. In contrast, significant reductions in integrin β1, integrin β3, IL-8, IL-6, and IL-1β levels were noted at the 24 h LPS treatment (Figure 6). Additionally, TNF-α levels were increased at 4 and 24 h, and DICAM overexpression decreased TNF-α. These findings highlight the role of DICAM in modulating the cellular milieu during LPS-mediated AKI, potentially emphasizing its significance in preserving cell junctional integrity and reducing inflammatory processes.

### 3.4. Association between DICAM Expression and Apoptosis in HK-2 Cell Injury

To investigate the association between DICAM expression and cell death in HK-2 cell injury, HK-2 cells were treated with DICAM siRNA to reduce DICAM expression, followed by exposure to H_2_O_2_ to induce oxidative stress (Figure S2a). In addition, HK-2 cells were genetically modified to overexpress DICAM using an adenoviral vector (Ad-DICAM) and then treated with LPS to induce stress (Figure S2b). A decrease in Bcl2 mRNA levels, an anti-apoptotic gene, was observed in the DICAM siRNA group, suggesting increased apoptosis. Caspase 3 activity, a marker of apoptosis, was significantly increased in the DICAM siRNA group, supporting this increased apoptosis. Conversely, in LPS-induced injury, caspase 3 activity was reduced in the Ad-DICAM group with DICAM overexpression compared to the control (Ad-GFP), confirming that DICAM overexpression reduces cell death. The results of both experiments consistently support a protective role of DICAM against apoptosis in HK-2 cell injury.

## 4. Discussion

This study aimed to explore the role and implications of DICAM in renal cell injury, particularly in the context of oxidative and inflammatory stressors. We demonstrated the protective role of DICAM in acute renal cell injury and its potential as a predictor of AKI. Recent findings have underscored the versatile role of DICAM in various inflammatory conditions. DICAM demonstrates anti-inflammatory effects in diverse contexts, including neuroinflammation, gastrointestinal disturbances, osteoarthritis, osteoclast differentiation, vascular pathologies, and macrophage activation [10,11,12,13]. We confirmed for the first time that DICAM also plays an anti-inflammatory role in kidney injury in cellular experiments. The results of previous studies and ours highlight the consistent anti-inflammatory properties of DICAM and its potential as a therapeutic target for multiple inflammatory diseases, including AKI.

Our results highlight a marked increase in DICAM expression following H_2_O_2_ and LPS treatments in HK-2 cells. It appears that DICAM significantly responds to oxidative and inflammatory injuries, suggesting its potential involvement in cellular defense mechanisms. A previous study has demonstrated that oxidative stress is a critical player in AKI onset and progression, with H_2_O_2_ being a widely recognized representative of such oxidative agents [16]. Similarly, LPS, a Gram-negative bacteria component, can simulate sepsis-induced kidney injury with a powerful inflammatory response [17]. This increased expression of DICAM is consistent with previous findings of an increased expression of DICAM upon the induction of kidney injury in animal models of cisplatin-induced AKI [14]. This suggests that DICAM expression is increased in the kidney upon the induction of AKI, regardless of the cause. Furthermore, similar to the increase in DICAM expression in AKI model and other inflammatory diseases, including colitis and neuroinflammation [12,13], DICAM upregulation following the induction of kidney injury could be interpreted as a cellular response aimed at mitigating injury. Considering the results of the present study and other findings regarding the role of DICAM, further exploration into the signaling pathways influenced by DICAM could provide deeper insights into its mechanistic role in AKI. Understanding these pathways could reveal potential intersections with other known renal injury mechanisms, offering a more integrated view of kidney pathophysiology.

The suppression of occludin, a tight junction protein essential for maintaining epithelial integrity, following H_2_O_2_ and LPS treatments, coupled with the concurrent increase in DICAM, suggest a compensatory mechanism. In kidney injury, occludin depletion weakens epithelial integrity, thereby making cells more vulnerable to injury, and enhanced DICAM may reinforce cell adhesion, thereby minimizing damage. Furthermore, increased expression of DICAM was associated with a reduction in oxidative stress factors and inflammatory markers, suggesting that it may have direct antioxidant or anti-inflammatory activity. Our hypothesis has been supported by the results of knockdown and overexpression experiments. Following DICAM knockdown, a significant increase in inflammation-related markers including NOX4, IL-1β, and IL-6 was observed in H_2_O_2_ and LPS treatments. NOX4 is a remarkable contributor to oxidative stress in renal cells, and IL-1β and IL-6 are potent inflammatory cytokines [18,19]; these markers are all known to play a significant role in renal cell injury in AKI [20]. The changes in these markers indicate that DICAM serves as a positive regulator of inflammation in its basal state. In addition, DICAM overexpression led to a noticeable increase in occludin levels and a decrease in inflammation-related markers, including integrin β1, integrin β3, IL-6, and IL-1β, following LPS treatment. This further strengthens the hypothesis of a protective role of DICAM in renal cells during AKI.

Moreover, our hypothesis has been supported by the results of knockdown and overexpression experiments. Following DICAM knockdown, a significant increase in inflammation-related markers including NOX4, IL-1β, and IL-6 was observed in H_2_O_2_ and LPS treatments. NOX4 is a remarkable contributor to oxidative stress in renal cells, and IL-1β and IL-6 are potent inflammatory cytokines [18,19]; these markers are all known to play a significant role in AKI [20]. The changes in these markers indicate that DICAM serves as a positive regulator of inflammation in its basal state. In addition, DICAM overexpression led to a noticeable increase in occludin levels and a decrease in inflammation-related markers, including integrin β1, integrin β3, IL-6, and IL-1β, following LPS treatment. This further strengthens the hypothesis of a protective role of DICAM in renal cells during AKI. However, it is difficult to determine the specific role or action of DICAM based on this study, and further research is needed.

Further, we have confirmed the potential of DICAM as an early predictor of AKI. Interestingly, our current findings suggest that DICAM expression precedes that of NGAL in AKI. NGAL has emerged as an effective biomarker for early AKI diagnosis, reflecting renal cell damage, in various studies [21,22]. NGAL is a useful indicator in AKI because early and rapid NGAL elevation during AKI offers clinicians a valuable tool for prompt detection and subsequent therapeutic intervention [23]. This consistent accelerated DICAM expression across various AKI etiologies such as ischemia and inflammation provide a compelling suggestion. Our results suggest that DICAM has the potential to effectively predict AKI in its early stages, surpassing the performance of NGAL. We have measured DICAM in blood samples from patients with severe AKI, confirmed that DICAM is elevated compared to normal individuals without AKI, and are conducting further analysis. An early diagnosis of AKI using DICAM is expected to improve AKI management with more timely intervention and thus improve AKI outcomes, but further studies in AKI patients are needed to prove this.

**Figure 7:**
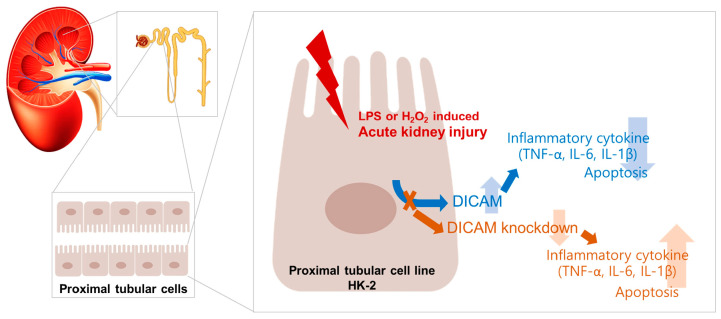
A summary of the results from the present study. LPS or H_2_O_2_ injury induced increased expression of DICAM in human proximal tubular epithelial cells. Increased DICAM suppressed inflammatory cytokine expression such as TNF-α, IL-6, and IL1β. When DICAM was knockdown and exposed to H_2_O_2_ injury, the inflammatory response was increased in proximal tubular cells. Abbreviations: dual immunoglobulin domain-containing cell adhesion molecule; LPS, lipopolysaccharide; H_2_O_2_, hydrogen peroxide; TNF-α, tumor necrosis factor-α; IL-6, interleukin 6; IL-1β, interleukin-1 beta.

Figure 7 summarizes the results of the study. Experiments were performed on proximal tubular cells, which are commonly injured in AKI. The expression of DICAM in human proximal tubular epithelial cells increased from the early phase of the injury with LPS and H_2_O_2_, and DICAM played a role in suppressing inflammation, which reduced cell injury such as apoptosis. The knockdown of DICAM increased the inflammatory response and increased apoptosis, supporting these findings.

Although our study provides a novel protective role of DICAM in renal cell damage in AKI and its potential as an early predictive biomarker, several limitations must be acknowledged. First, the comparisons between DICAM and the established AKI biomarker (NGAL) are based on observational findings; therefore, to validate these initial observations, prospective studies are needed. Second, although the anti-inflammatory properties of DICAM across various diseases suggest a common underlying mechanism, distinct pathophysiological mechanisms were not explored in our study. Finally, our in vitro study results were not confirmed in human or animal studies. Confirming our results through additional in vivo studies is necessary.

In conclusion, our findings highlight the potentially critical role of DICAM in relation to renal tubular cell injury. Its behavior in response to oxidative and inflammatory stress and its effects when manipulated suggest its significance as a protective agent. Additionally, DICAM was observed to increase during the early stage of renal tubular cell injury, indicating its potential as an early diagnostic marker for AKI. These functions of DICAM highlight its importance in renal cell injury from various causes and propose its potential as a diagnostic marker and a therapeutic target.

## Data Availability

The data that support the findings of this study are available from the corresponding author upon reasonable request. The data are not publicly available due to ethical restrictions.

## Supplementary Materials

The following supporting information can be downloaded at https://www.mdpi.com/article/10.3390/cimb46030115/s1, Figure S1: HK-2 cell viability test after H_2_O_2_ and LPS treatment; Figure S2: Association of DICAM expression and apoptosis in HK-2 cell injury.
