# Supplementary material for: Dual Immunoglobulin Domain-Containing Cell Adhesion Molecule Increases Early in Renal Tubular Cell Injury and Plays Anti-Inflammatory Role
Source: Curr Issues Mol Biol. 2024 Feb 26;46(3):1757–67. doi: 10.3390/cimb46030115 (PMC10969420; doi:10.3390/cimb46030115)
Supplement: Supplementary file 1 [file cimb-46-00115-s001.zip › cimb-2861202-supplementary.pdf]

Figure S1. HK-2 cell viability test after H_2O_2 and LPS treatment.

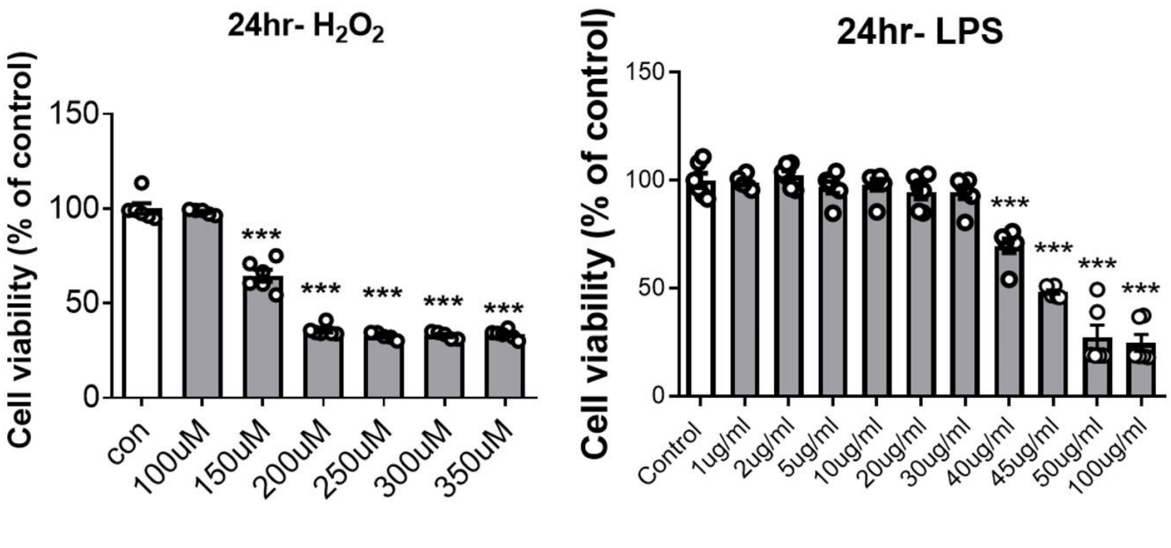

*** $p < 0.001$ indicate statistical significance.

Figure S2. Association of DICAM expression and apoptosis in HK-2 cell injury.

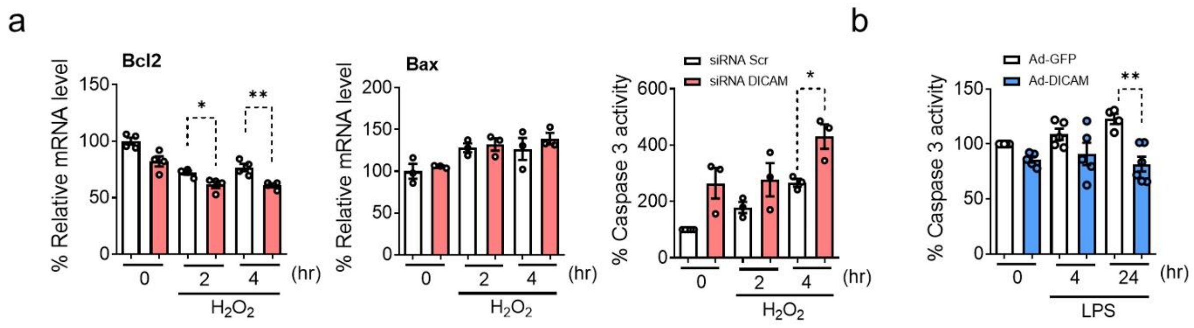

* $p < 0.05$, ** $p < 0.01$ indicate statistical significance.
